# Prevalence of depressive symptoms and correlated factors among pregnant women during their second and third trimesters in northwest rural China: a cross-sectional study

**DOI:** 10.1186/s12884-021-04340-0

**Published:** 2022-01-16

**Authors:** Fang Chang, Xin Fan, Yi Zhang, Bin Tang, Xiyuan Jia

**Affiliations:** 1 Center for Experimental Economics in Education, Shaanxi Normal University, Xi’an, 710119 Shaanxi China; 2 Department of Economics and Finance, City University of Hong Kong, Hong Kong, China

**Keywords:** Depression symptoms, Pregnant women, Folic acid, Rural China

## Abstract

**Background:**

The depression mood during their second and third trimesters has a negative impact on both the mother and her child. Compared with pregnant women in urban areas, rural pregnant women who are in more disadvantaged situation may have more serious psychological problems. Particular, many rural pregnant women had internal migrant work experience during pregnancy in rural China. It is thus necessary to study the prevalence of depressive symptoms and correlated factors among Chinese northwest rural pregnant women.

**Methods:**

This study was conducted from October 2017 to April 2018 and surveyed 1053 pregnant women in the middle and late stages of pregnancy who were registered in rural areas, gave informed consent and did not suffer from cognitive impairment or severe mental illness. Depressive symptoms were evaluated by the Chinese Version of the Short Depression Anxiety and Stress Scale (DASS-C21). Demographic characteristics, pregnancy characteristics and family factors were obtained through structured questionnaires. This study employed multiple factor logistic regression to analyze the relationship between depressive symptoms and their correlates.

**Results:**

The prevalence of depressive symptoms among pregnant women during their second and third trimesters was 16.14% (95%CI 13.92%-18.36%). Higher education levels (OR = 0.50; 95%CI 0.29–0.85) and taking folic acid (OR = 0.59; 95%CI 0.39–0.89) reduced the risk of depression symptoms. The family receiving rural welfare (OR = 1.69; 95%CI 1.04–2.75), migration for work (OR = 1.95; 95%CI 1.03–3.71) and living with both parents and parents-in-law (OR = 2.55; 95%CI 1.09–5.96) increased the risk of depressive symptoms.

**Conclusions:**

The prevalence of depressive symptoms among pregnant women during their second and third trimesters in Northwest rural China was 16.14% that was nearly 4 percentage points higher than the average survey result of the pregnant women in developed countries and was higher than the findings in Chinese urban areas. To prevent depression symptoms, it’s essential to early screen and provide folic acid for free when antenatal examination. Moreover, maternal examination files should be established so that wo pay attention to the psychological status of pregnant women who were with low education levels, poor family economic situations, excessive parental burden and who had been migrant workers.

## Strengths and limitations of this study

- This is a cross-sectional study with a large sample in northwest rural China.
- A limitation of this study is that the sample is not entirely randomly selected.
- We do not measure several important factors related to depressive symptoms such as a history of depression episodes, history of psychological trauma and family history of mental illness.

## Background

While pregnancy is a normal psychosocial event for women, it can also lead to large psychological changes, and pregnant women may go through a series of depressive behaviors [1]. The depression mood during pregnancy has become a serious problem, and recent evidence confirms that it can have a negative impact on both the mother and her child [2, 3]. Research has shown that about 82.76% of women with depressive symptoms that occur during pregnancy tend to develop postpartum depression [4]. Depression threatens the health, marriage and family relations of pregnant women [5]. In addition, it also seriously affects pregnancy outcomes like prematurity of birth, birth weight, and head circumference [6, 7], and may influence the mood, cognitive ability and the language and behavior development of the child [2]. Depression also has a large impact on mother-child relationships [8].

Depression may change over the course of pregnancy, with symptoms that vary between the first trimester (< 14 weeks), second trimester (14–27 weeks) and third trimester (> 27 weeks) [9]. Incidence of depression is particularly high during the second and third trimesters [3]. Research reveals that the average incidence of depression in the second and third trimesters is nearly 5 percentage points higher than in the first trimester [3, 6, 10–12]. With the rapid development of the fetus in the second and third trimesters, pregnant women experience many physical and psychosocial changes, which can increase the risk of depression [1]. Therefore, it is of great significance to consider the antenatal mental health status of mothers and to determine potentially correlated factors of antenatal mental health by studying pregnant women during their second and third trimesters.

The factors that affect depression during pregnancy are mainly divided into three categories: personal, family and social. Personal factors for depression in women mainly include four domains: (1) demographic characteristics; (2) knowledge; (3) attitudes and behaviors, (4) obstetric characteristics. Family factors include the support and life events associated with spouses, parents and parents-in-law. Social factors mainly refer to interactions with medical staff and friends during pregnancy [13]. Within these factors, younger age, low education level, dysfunctional family relationships, lack of social support [14, 15], first pregnancy, irregularity of checkups during pregnancy, lack of knowledge about pregnancy [16], lack of pregnancy training and lower family income increase the risk of depression in pregnant women [6, 10].

Previous studies have mostly focused on pregnant women in urban area and rural areas [17–25], but China rural pregnant women are not given enough attention [26]. Due to greater expenditure with transport, poorer transport infrastructure and longer travel times [27], fewer options for childcare provision [28], rural pregnant women who are in more disadvantaged situation may have more serious psychological problems than urban pregnant women [26, 29, 30], such as later initiation of prenatal care [31], as well as higher chances of a small-for-gestational-age birth and low birth-weight [32]. It is thus necessary to study the symptoms of depression in China rural pregnant women. Meanwhile, among rural migrant workers, the number of women reached two fifths [33]. When they are of marriageable age or pregnant, they have to return home. Later, it was difficult to go out for work because of things such as childcare [34, 35]. Compared with pregnant women in urban areas, it was particular that many rural pregnant women had internal migrant work experience during pregnancy in rural China (In this study, the proportion of pregnant women who had been migrant workers was 88.22% in rural China). This study attempts to explore the effect and relevant mechanisms that internal migrant work experience on the risk of depression symptoms. Because the rural area of northwest China is one of the areas with a largest number of people going out for work [36], the study aims to describe the prevalence and correlates of depressive symptoms in women in their second and third trimesters in Northwest rural China. The results of this study may provide a basis for reducing the risk of pregnancy depression in rural areas of China and promoting the healthy development of babies.

## Method

### Design and participants

This study followed a cross-sectional design, surveying pregnant women in their second and third trimesters in rural areas of 7 counties in Northwestern China, and was conducted between October 2017 and April 2018. The sampling method is as follows. Firstly, to achieve a representative sample of northwest rural areas, 5 nationally-designated poverty counties and 2 non-poverty counties, as designated by their development level in 2017, were selected for inclusion. Secondly, as the county seat is often better developed than the rest of the county, and we wish to focus on rural areas, we excluded all county seats from our sample, and included all other towns in each county in our sample. After this selection process, 206 towns were included in our sample.

**Fig. 1:**
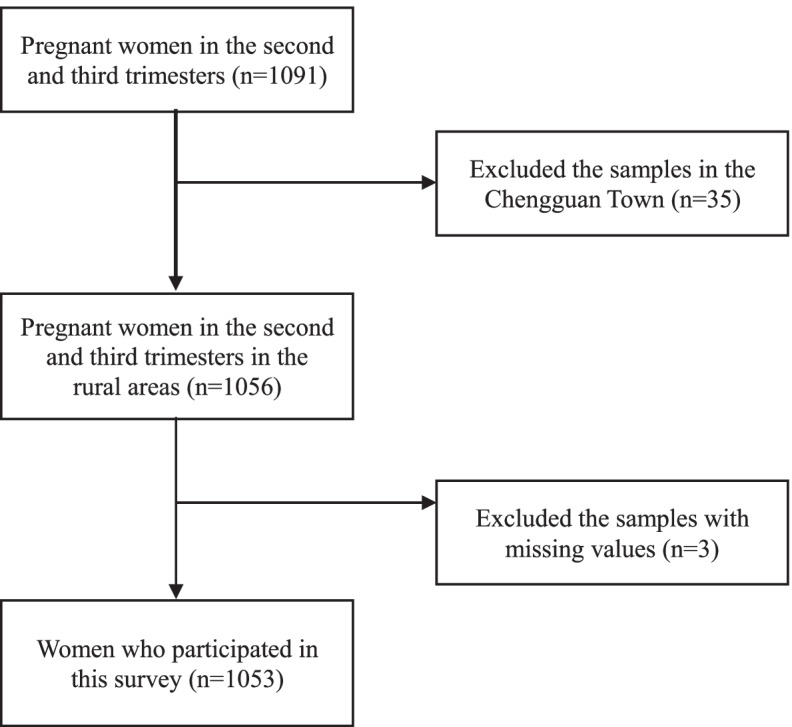
Sample flow chart

We included all pregnant women in their second and third trimester (gestational frame: 20–32 weeks) who had lived in a town in our sample for more than 6 months and had given signed informed consent. We excluded women with cognitive disorders, severe mental illnesses or other serious diseases and those with missing reported data values. The sampling process is shown in Fig. 1.

The sample size was estimated using the following equation. Previous research has shown that the incidence of depression is around 11.7% in rural Northwestern China [37]. Setting the significant level α at 0.05 and the permissible error quantity d at 0.02, the minimum theoretical sample size is 992. From our sample, 1053 pregnant women filled out paper questionnaires, and the results were further analysed.

$$\mathrm{n}={Z}_{1-\frac{\alpha }{2}}^2p\left(1-p\right)/{d}^2$$

### Procedure

Prior to administering the survey, all investigators underwent a one-week standardized training session and were familiarized with the investigation process via a pre-survey. After obtaining informed consent from study participants, the study team collected participant data. To ensure the authenticity and reliability of the survey data, a standardized script was used to conduct face-to-face structured interviews with the subjects where a questionnaire was administered. After the completion of the questionnaire, three investigators cross-checked participant answers to ensure accuracy.

### Measurement

#### Basic information

The Basic Information Questionnaire (Table 1) was a self-report questionnaire that was used to obtain the demographic information of participants, including their age, education level, occupation and physical health. Pregnancy characteristics included gestational time (unit: weeks), parity (1, ≥2), whether the pregnancy test was on time, whether the mother participated in pregnancy training and whether the mother was taking folic acid. Family factors included family economic condition, whether the mother was a migrant worker, whether the husband was a migrant worker and whether the mother lived with parents or parents-in-law.

**Table 1:** Assignment of independent variables

Characteristic	Assignment
**Demographic characteristics**	
Age	< 30 = 0; ≥30 = 1
Education level	Primary school = 0;
	Junior middle school or above = 1
Occupation (whether there is a job)	No = 0; Yes = 1
State of physical health	Unhealthy = 0; Healthy = 1
**Pregnancy characteristics**	
First child	No = 0; Yes = 1
Regular antenatal examination	No = 0; Yes = 1
Participate in pregnancy training	No = 0; Yes = 1
Taking folic acid	No = 0; Yes = 1
**Family factors**	
Family economic situation	No basic living allowance = 0;
	With basic living allowance = 1
Once being internal migrant worker	No = 0; Yes = 1
Be internal migrant worker (husband)	No = 0; Yes = 1
Whether to live with elders	No living with elders = 0;
	Live with parents-in-law = 1;
	Live with parents = 2;
	Live with parents-in-law and parents =3

#### Outcome measurement

Depressive symptoms during pregnancy were assessed by the Chinese version of the Short Depression Anxiety and Stress Scale (DASS-C21) [38]. With reference to the traditional Chinese version compiled by Mouse in Hong Kong, China, and the original English version compiled by Lovibond [39, 40], the simplified Chinese version DASS-C21 was adapted for the language habits of mainland adult residents [38]. Both in China and abroad, Cronbach α of DASS-C21 scale was greater than 0.82 and the loads of the items on their corresponding factors were all 0.39 ~ 0.79. This suggest that the scale had good reliability and validity and could be used as an effective tool in evaluating depression, anxiety and stress in adult residents [38–40]. The DASS-C21 scale included 21 items, consisting of 3 subscales of depression risk, anxiety, and stress. Each subscale had 7 items. Item scores ranged between 0 (not at all) and 3 (very consistent). Each subscale score is multiplied by 2 for a final maximum total score of 42. A higher score denotes a higher frequency of depressive symptoms within the past week.

In this study, the depression subscale (see Table 2 for each item) was used to assess the severity of depressive symptoms in sample participants. According to the DASS grading standards, degrees of depression were divided into 5 levels: 0–9 was normal, 10–13 was mild, 14–20 was moderate, 21 ~ 27 was severe and 28 and above was serious [38, 41]. In this study, the “normal” level was classified as having no depressive symptoms, and the other 4 levels were classified as having depressive symptoms. In short, if a participant scored greater than or equal to 10, they were determined to experience depressive symptoms [42]. The Cronbach’s α of the depression subscale used in this study was 0.83. The loads of the of the depression subscale’s items on their corresponding factors were all 0.48 ~ 0.74.

**Table 2:** Depression symptoms measurement indicators

1	I couldn’t seem to experience any positive feeling at all
2	I found it difficult to work up the initiative to do things
3	I felt that I had nothing to look forward to
4	I felt down-hearted and blue
5	I was unable to become enthusiastic about anything
6	I felt I wasn’t worth much as a person
7	I felt that life was meaningless

### Statistical analysis

Stata15.1 was used to analyze all data in this study. Continuous and categorical variables were converted into dummy variables by referring to the literature, and the first category of each variable was used as the reference group during regression analysis. Depressive symptoms were analyzed as numbers and percentages. All characteristic variables (eg, demographic information, pregnancy characteristics and family factors) were analyzed as counts and percentages. To compare characteristics between groups with depressive symptoms and those without, the χ^2^ test was used for categorical variables. Finally, a multivariate binary logistic regression with ORs was used to analyze the correlates of depressive symptoms. All statistical tests were two-sided.

### Patient and public involvement

This study was conducted without patient and public involvement.

## Result

### Sample characteristics

The mean score of depressive symptoms (Table 3) for women in their second and third trimesters was 4.57 points. On average, women in their second and third trimesters in rural areas were within the normal score range. Among the 1053 samples analyzed, 607 (57.64%) participants were in their second trimester and 446 (42.36%) were in their third trimester. A total of 170 participants (16.14%) had depressive symptoms. As the depressive level increased, the number of women got depressive symptoms decreased. The proportions of depressive symptoms during the second and third trimesters were 16.47% and 15.70% respectively, showing that the rates of depressive symptoms were not insignificantly different between the second and third trimesters.

**Table 3:** Current status of depression symptoms in pregnant women

Depression level	Overall (*n* = 1053)	Second trimester (*n* = 607)	Third trimester (*n* = 446)
Normal	883 (83.86%)	507 (83.53%)	376 (84.30%)
Mild	84 (7.98%)	51 (8.40%)	33 (7.40%)
Moderate	68 (6.46%)	40 (6.59%)	28 (6.28%)
Severe	10 (0.95%)	4 (0.66%)	6 (1.35%)
Serious	8 (0.76%)	5 (0.82%)	3 (0.67%)
Total incidence	170 (16.14%)	100 (16.47%)	70 (15.70%)

Total incidence was equal to the sum of the incidence of pregnant women in “Mild”, “Moderate”, “Severe” and “Serious” levels

In terms of basic characteristics (Table 4), more than half of study participants were under 30 years old (77.11%). Most participants had a junior high school degree or above (91.36%), and less than a third had stable jobs (21.65%). Most considered themselves to be in good health (89.08%). A total of 40.17% were pregnant for the first time. Over half of participants (57.64%) had received regular prenatal examinations. Only 9.02% had participated in pregnancy training. Folic acid was taken by 83.10% of the participants.

**Table 4:** Basic Information of women and distribution of depression symptoms (*n* = 1053)

Characteristic	Depression symptoms		χ^**2**^	***P*** value
	Total n (%)	Yes n (%)		
**Demographic characteristics**				
Age			0.38	0.538
< 30	812 (77.11%)	128 (75.29%)		
≥ 30	241 (22.89%)	42 (24.71%)		
Education level			**11.36**	**0.001**
Primary school	91 (8.64%)	26 (15.29%)		
Junior middle school or above	962 (91.36%)	144 (84.71%)		
Occupation (whether there is a job)			3.21	0.073
Yes	228 (21.65%)	28 (16.47%)		
No	825 (78.35%)	142 (83.53%)		
State of physical health			**5.12**	**0.024**
Unhealthy	115 (10.92%)	27 (15.88%)		
Health	938 (89.08%)	143 (84.12%)		
**Pregnancy characteristics**				
First child			0.00	0.960
Yes	423 (40.17%)	68 (40.00%)		
No	630 (59.83%)	102 (60.00%)		
Regular antenatal examination			0.03	0.865
Yes	607 (57.64%)	99 (58.24%)		
No	446 (42.36%)	71 (41.76%)		
Participate in pregnancy training			3.43	0.064
Yes	95 (9.02%)	9 (5.29%)		
No	958 (90.98%)	161 (94.71%)		
Taking folic acid			**8.78**	**0.003**
Yes	875 (83.10%)	128 (75.29%)		
No	178 (16.90%)	42 (24.71%)		
**Family Factors**				
Family economic situation			**7.24**	**0.007**
With basic living allowance	107 (10.16%)	27 (15.88%)		
No basic living allowance	946 (89.84%)	143 (84.12%)		
Once being internal migrant worker			**4.34**	**0.037**
Yes	929 (88.22%)	158 (92.94%)		
No	124 (11.78%)	12 (7.06%)		
Be internal migrant worker (husband)			0.36	0.550
Yes	523 (49.67%)	88 (51.76%)		
No	530 (50.33%)	82 (48.24%)		
Whether to live with elders				
**Reference:** No living with elders	286 (27.16%)	46 (27.06%)		
Live with parents-in-law	637 (60.49%)	98 (57.65%)	0.07	0.787
Live with parents	99 (9.40%)	16 (9.41%)	0.00	0.986
Live with parents-in-law and parents	31 (2.94%)	10 (5.88%)	**5.03**	**0.025**

The significance of bold font is *P* < 0.05, and the results are statistically significant

Regarding family factors, 10.16% of families had received rural welfare. Most women (88.22%) were once being migrant workers. Almost half of participants’ husbands had been working away from home in the past year (49.67%). More than half of pregnant women lived with their parents-in-law (60.49%), while only 9.40% of them lived with their own parents. Only 2.94% of them lived with both parents-in-law and their parents.

Taking depressive symptoms (1 = Yes, 0 = No) as the dependent variable, the univariate analysis (Table 4) shows that education level, state of physical health, folic acid use, family economic situation, migrant work and living with parents or parents-in-law are significantly correlated with depressive symptoms during the second and third trimester (*P* < 0.05). No other characteristics exhibit a significant correlation with depressive symptoms.

### Multivariate regression analysis of depression symptoms for pregnant women

We included demographic characteristics, pregnancy characteristics and family factors in the multivariate regression analysis (Table 5). Multivariate logistic regression showed that in pregnant women, the odds of experiencing symptoms of depression in the second and third trimester were reduced by having a junior middle school degree or above (OR = 0.50; 95%CI 0.29–0.85) and taking folic acid (OR = 0.59; 95%CI 0.39–0.89). The odds of experiencing depressive symptoms were increased by the family receiving rural welfare (OR = 1.69; 95%CI 1.04–2.75), once being a migrant worker (OR = 1.95; 95%CI 1.03–3.71) and living with parents and parents-in-law (OR = 2.55; 95%CI 1.09–5.96).

**Table 5:** Multivariate regression analysis of depression symptoms for pregnant women

Variables	β	SE	OR(95%CI)	***P*** value
**Demographic characteristics**				
Age				
< 30	Reference			
≥ 30	0.08	0.223	1.09 (0.70–1.68)	0.713
Education level				
Primary school	Reference			
Junior middle school or above	**−0.70**	**0.277**	**0.50 (0.29–0.85)**	**0.011**
Occupation (whether there is a job)				
Yes	−0.30	0.230	0.74 (0.47–1.16)	0.188
No	Reference			
State of physical health				
Unhealthy	Reference			
Health	−0.30	0.258	0.74 (0.45–1.23)	0.253
**Pregnancy characteristics**				
First child				
Yes	0.11	0.189	1.11 (0.77–1.61)	0.564
No	Reference			
Regular antenatal examination				
Yes	0.08	0.176	1.08 (0.76–1.52)	0.666
No	Reference			
Participate in pregnancy training				
Yes	−0.48	0.367	0.62 (0.30–1.27)	0.188
No	Reference			
Taking folic acid				
Yes	**−0.53**	**0.207**	**0.59 (0.39–0.89)**	**0.011**
No	Reference			
**Family Factors**				
Family economic situation				
With basic living allowance	**0.52**	**0.249**	**1.69 (1.04–2.75)**	**0.036**
No basic living allowance	Reference			
Once being internal migrant worker				
Yes	**0.67**	**0.328**	**1.95 (1.03–3.71)**	**0.041**
No	Reference			
Be internal migrant worker (husband)				
Yes	0.02	0.177	1.02 (0.72–1.44)	0.903
No	Reference			
Whether to live with elders				
No living with elders	Reference			
Live with parents-in-law	0.03	0.203	1.03 (0.69–1.53)	0.901
Live with parents	0.06	0.330	1.06 (0.55–2.02)	0.862
Live with parents-in-law and parents	**0.94**	**0.434**	**2.55 (1.09–5.96)**	**0.031**

The bold font indicates that the results are statistically significant

## Discussion

### Prevalence of depressive symptoms

As a generally accepted global public health and social problem, depression is characterized by a significant and lasting state of low mood [43]. From our sample of 1053 pregnant women in their second and third trimesters in rural Northwestern China, we found that the incidence rate of depression symptoms was 16.14% (95%CI 13.92%-18.36%), which was consistent with 15.6% on average in developing countries, but nearly 4 percentage points higher than the average survey result of the pregnant women in developed countries in the second and third pregnancy [44]. Meanwhile, it was higher than the survey findings in urban areas on Chinese six provinces including Hebei, Liaoning, Fujian, Hunan, Sichuan and Yunnan province [17, 37]. Depressive symptoms in pregnant women during their second and third trimesters may therefore be a more serious problem in Northwestern China compared to other areas, and should be taken into consideration for future research.

### Correlates of depressive symptoms during the second and third trimesters

#### Demographic characteristics

As for personal characteristics, this study showed that age and occupation have no significant impact on the depressive symptom rural women during their second and third trimesters. In contrast, previous research using urban samples found that older women had more psychological issues since they played more important roles at work and feared that child birth would affect their career prospects [10]. In contrast to urban women, rural women mainly engaged in agriculture, self-run industry and commerce and internal migrant work, so pregnancies at an older age might seldom affect the career prospects of women in rural areas and thus age did not influence symptoms of depression.

In addition, higher levels of education during pregnancy imply that the mother would be more active in acquiring knowledge about pregnancy and solving problems, which would help to relieve psychological pressure [45, 46]. Similar to the previous studies, educational level significantly reduced depressive symptoms in the multivariate regression analysis.

This study found no significant link between health status and depressive symptoms. The existing studies indicated that pregnant women who had serious injury, were with pregnancy syndrome and other physical health conditions may easily bear adverse psychological outcomes [11]. This disagreement with previous research should be a consideration for future studies.

#### Pregnancy characteristics

In terms of pregnancy-related factors, whether the mother had previous pregnancies, whether the mother participated in a pregnancy checkup on time, and whether the mother participated in pregnancy training were not significantly related to the risk of depressive symptoms. From the nonsignificant coefficients of the variable whether the mother had previous pregnancies, we find that the odds of experiencing depressive symptoms were increased if the pregnant women were primipara, which is consistent with other studies [47, 48]. Probably because that primiparas were relatively unfamiliar with the physiological changes during pregnancy, and the fear of the unknown will increase the depression emotions of pregnant women [49]. In addition, pregnant women who took folic acid during pregnancy had a lower risk of depressive symptoms, consistent with previous international research [50, 51]. Calcium, iron, zinc and folic acid are the most popular supplements during pregnancy, and they are beneficial to the health of the pregnant woman and the development of the fetus [52–55]. In this paper, only taking folic acid was statistically significant (The result of calcium, iron and zinc are not shown). Probably because Chinese government only provides rural women folic acid for free, but not calcium, iron and zinc [56]. Hence, taking folic acid can improve health without increasing family’s financial burden, which helps reduce the risk of depression symptoms for pregnant women. In the study, the rate of taking folic acid during pregnancy among pregnant women in northwest rural areas was 83.10%, lower than 90.08% in developed regions in China [57]. To alleviate depressive symptoms, the distribution of folic acid to rural women of childbearing age should be further strengthened.

#### Family factors

Turning to family factors, this study showed that poor family economic situations was related to higher risk for depression in pregnant women. Childbirth is a relatively large economic expenditure for rural families. As the expected delivery date approaches in the second and third trimesters, pregnant women in families with financial hardship were overly worried about not being able to provide a good nurturing environment for their children in the future, bringing psychological distress [6].

In our study, the proportion of pregnant women who had been migrant workers was 88.22%, a factor that increased risk of depressive symptoms during the second and third trimesters. The possible reason was that the experience of being internal migrant workers had broadened their horizons and increased their economic income, so that women’s value was recognized. They had a stronger willingness to create a better life for their unborn children and also increased the expectation for their fetuses. However, the pressure of traditional gender division of labor makes women stayed at home to have children. It was difficult for them to realize their self-worth and expectations [34, 35]. Therefore, they might be more prone to depression.

Previous research shows that good group relationships (such as family, friends, etc.) help to reduce the risk of depression symptoms in pregnancy [58–61], but the results of this study were different. First, the migration status of the husband had no significant correlation with the depression symptoms. A possible explanation for this was that being a migrant worker could significantly improve the economic situation of the family [62], and bring a positive impact on pregnant women. At the same time, the temporary absence of a husband would force pregnant women to bear the full burden of a household and prevent emotional communication, leading to depressive symptoms [45, 63–65]. These positive and negative effects may cancel each other out to some extent. Second, living with only parents or parents-in-law was not correlated with symptoms of depression, however there was a significant correlation when lived with both parents and parents-in-law, a departure from existing studies. Looking at possible explanations, living with elders is one method of support during pregnancy given the traditional pension system in rural China [66], but intergenerational differences in the way of coping with problems make it difficult for elders to effectively help pregnant women reduce their risk of depression [67]. Currently, many pregnant women do not have siblings [11], and increasingly choose the “two-headed” living model (lived with both parents and parents-in-law), a sign that the husband and wife respect each other and have equal status. Pregnant women who live with both sets of parents need to bear the heavy burden of caring for their parents, which greatly increases their depressive tendencies [68].

## Conclusion

Compared with urban areas and developed countries, the prevalence of depression symptoms among pregnant women in rural areas of northwest China is higher. Pregnant women who had low education levels, poor family economic situations, excessive parental burden and had been migrant workers tended to have a higher risk for depressive symptoms. Certain health care during pregnancy, such as taking folic acid, might be an effective way to reduce depressive symptoms. These findings suggested that When antenatal examination, medical institution should add mental health screening tools in pregnancy examination among pregnant women during their second and third trimesters in northwest rural China to early detect and provide folic acid for free. Meanwhile, medical institutions also should establish maternal examination files that include pregnant women’s information about education levels, family economic situations, the number of supporting elderly people and whether had been migrant workers. When implementing maternal healthcare in medical institutions, we should pay attention to the psychological status of pregnant women who were with low education levels, poor family economic situations, excessive parental burden and had been migrant workers, and provide them with targeted maternal health nursing.

## Data Availability

The datasets used and/or analyzed during the current study are available from the corresponding author on reasonable request.

## Abbreviation

DASS-C21: The Chinese Version of the Short Depression Anxiety and Stress Scale
